# Further evidence for causal *FAM20A* mutations and first case of amelogenesis imperfecta and gingival hyperplasia syndrome in Morocco: a case report

**DOI:** 10.1186/1472-6831-15-14

**Published:** 2015-01-30

**Authors:** Imane Cherkaoui Jaouad, Mustapha El Alloussi, Siham Chafai El alaoui, Fatima Zahra Laarabi, Jaber Lyahyai, Abdelaziz Sefiani

**Affiliations:** Centre de Génomique Humaine, Faculté de Médecine et de Pharmacie, Université Mohammed V, Rabat, Morocco; Département de Génétique Médicale, Institut National d’Hygiène, Rabat, Morocco; Service d’odontologie pédiatrique, Faculté de médecine dentaire, Université Mohammed V, Rabat, Morocco

**Keywords:** Amelogenesis imperfecta, FAM20A, Gingival hyperplasia

## Abstract

**Background:**

Amelogenesis imperfecta represents a group of developmental conditions, clinically and genetically heterogeneous, that affect the structure and clinical appearance of enamel. Amelogenesis imperfecta occurred as an isolated trait or as part of a genetic syndrome. Recently, disease-causing mutations in the *FAM20A* gene were identified, in families with an autosomal recessive syndrome associating amelogenesis imperfecta and gingival fibromatosis.

**Case presentation:**

We report, the first description of a Moroccan patient with amelogenesis imperfecta and gingival fibromatosis, in whom we performed Sanger sequencing of the entire coding sequence of *FAM20A* and identified a homozygous mutation in the *FAM20A* gene (c.34_35delCT), already reported in a family with this syndrome.

**Conclusion:**

Our finding confirms that the mutations of *FAM20A* gene are causative for amelogenesis imperfecta and gingival fibromatosis and underlines the recurrent character of the c.34_35delCT in two different ethnic groups.

## Background

Amelogenesis imperfecta (AI) represents a group of developmental conditions, clinically and genetically heterogeneous, which affect the structure and clinical appearance of enamel, typically in both the primary and permanent dentition [1].

AI can be sub-classified on the basis of the mode of inheritance, the phenotype, and if known, the molecular cause of the enamel defects [2]. The phenotypic classification takes into account the two major steps of the enamel formation. AI can be divided into the hypomineralized AI which has a near-normal enamel matrix volume that is not normally mineralized, hypocalcified AI with a soft enamel which can be scraped away by hand, and hypomature AI with a fragile enamel and prone to fracture. In contrast, in hypoplastic AI there is failure of enamel matrix formation. Various enamel defects (both hypoplastic and hypomineralised) may coexist in the same patient and even in the same tooth.

Mutations in genes encoding enamel matrix proteins (AMELX, MIM 30039; ENAM, MIM 606585), enamel matrix proteolytic enzymes (KLK4, MIM 603767; MMP20, MIM 604629), an ion transporter (SLC24A4, MIM 609840), a putative crystal nucleator (C4orf26, MIM614829), and integrins and laminins (ITGB4, MIM 147557; LAMB3, MIM 150310; ITGB6, MIM 147558) have been identified in different forms of AI [3–11]. Other mutations have been reported in *FAM83H* (MIM 611927) and *WDR72* (MIM 613214), which are of unknown functions [12, 13].

AI exists in isolation or in association with other symptoms in syndromes, such as tricho-dento-osseous syndrome (MIM 190320), due to *DLX3* mutations, an autosomal-dominant disorder characterized by curly hair at birth, enamel hypoplasia, taurodontism, and thick cortical bone, and *CNNM4* mutations resulting in Jalili syndrome, which comprises autosomal recessive cone-rod dystrophy and AI (MIM#217080).

In 2008, Martelli et al. described four patients from a consanguineous family, with gingival hyperplasia and dental abnormalities including generalized thin hypoplastic amelogenesis imperfecta, with an autosomal recessive inheritance (AIGFS; MIM#614253) [14].

Recently, O’Sullivan et al. identified, by whole exome analysis, a homozygous nonsense mutation in exon 2 of the *FAM20A* gene [15], segregating with the syndrome in the family reported by Martelli-Junior et al. in 2008. More recently, Cho et al. identified homozygous and compound heterozygous mutations in the *FAM20A* gene in hypoplastic amelogenesis imperfect [16]. *FAM20A* can cause AIGFS and enamel renal syndrome (ERS; MIM# 204690).

In this study, we report the first Moroccan case showing AIGFS caused by mutations in *FAM20A* gene.

## Case presentation

**Figure 1:**
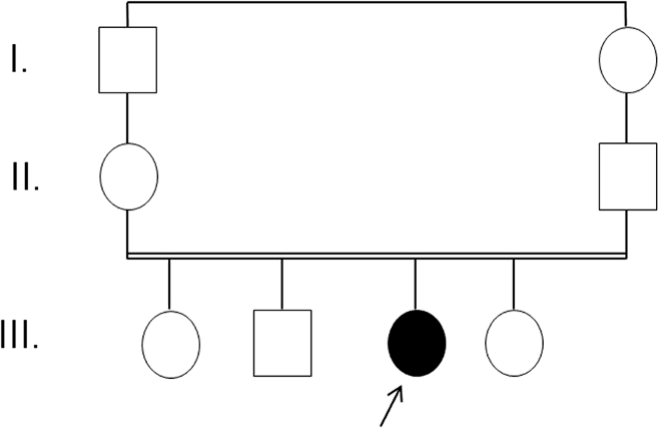
**Pedigree of the studied family.** Filled symbols represent affected individuals and open symbols represent unaffected individuals.

**Figure 2:**
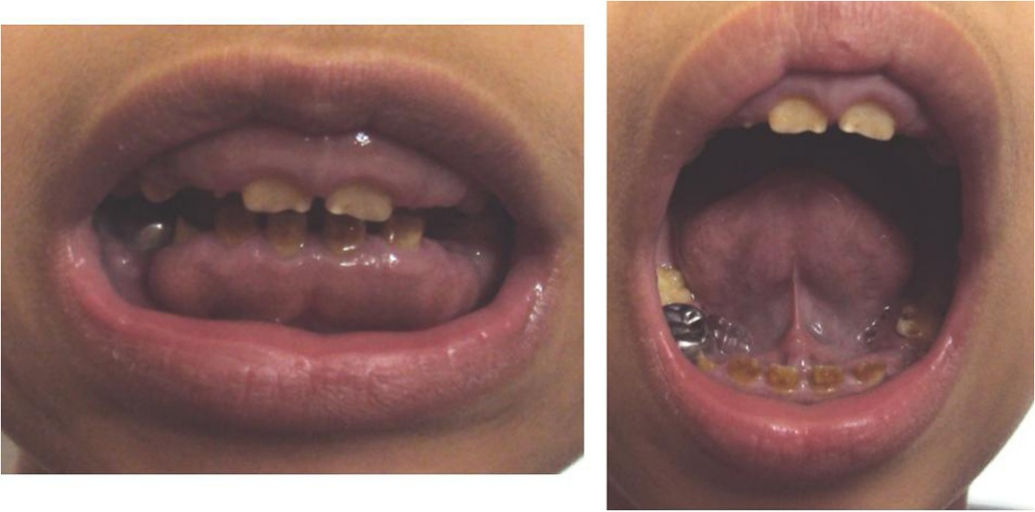
**Oral photographs of the proband at the age of 11 years.**

**Figure 3:**
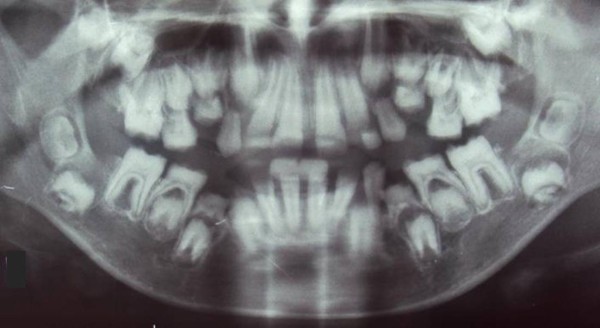
**Panorex radiograph of the proband.**

We investigated a sporadic index case of Moroccan origin, referred to the department of pediatric dentistry for dental treatment. The Index case was an 11 years old girl, the third child of a healthy consanguineous couple. She was the only affected member in the family (Figure 1). Her major complaints were the poor appearance of her teeth and the inability to chew properly, without other serious diseases. Dental examination of the patient revealed that primary and permanent dentition are affected by hypoplastic amelogenesis imperfecta, the crowns were short, yellow-brown, and covered with either a little or no enamel. The proband has also a generalized severe gingival hyperplasia, with a supra-incisive diastema (Figure 2). The panorex showed a secondary retention of four first molars, a pulpal obliteration of the right and left permanent superior molar and absence of enamel on all the teeth (Figure 3). On the other hand, the ultrasound analysis of the proband’s kidneys was negative for nephrocalcinosis, furthermore the proband doesn’t have any other pathological signs and no mental delay was noted. Thus, the diagnosis of AIGFS was made.

For the treatment plan, the four primary molars were extracted. The teeth were restored by stainless steel crown in all second primary molar and four first permanent teeth. Furthermore, gingivectomy was achieved in all sectors.

Venous blood samples were collected from the patient and his parents. Written informed consent was obtained before genetic testing from the parents. Proband was subject to mutational screening of the entire coding sequence of *FAM20A* (MIM *611062; GenBank NM_017565). Primers for amplification and sequencing of all eleven exons of human *FAM20A* and their flanking intronic regions were designed by using the University of California, Santa Cruz (UCSC) genomic browser (http://genome.ucsc.edu/). Sanger sequencing was done with dye terminator chemistry (ABI Prism BigDye v3.1) and run on the automated sequencer Applied Biosystems Prism 3100 DNA Analyzer. The obtained sequences were aligned to the reference genome (GRCh37/hg19) using DNA Variant analysis software (Mutation Surveyor® software). The established variants were cross-checked with the « clinvar » database (http://www.ncbi.nlm.nih.gov/clinvar/) which theoretically contains all genes variations.

Upon *FAM20A s*equencing, we detected a homozygous mutation c.34_35delCT (p.Leu12Alafs*67) in exon 1 of the gene. This mutation is predicted to result in frame shifts and premature termination codons. As expected, the parents turned out to be heterozygous carriers.

## Discussion

This case report represents an infant who presented with typical clinical features of AIGFS.

AIGFS is a rare autosomal recessive disease characterized by mild gingival hyperplasia and dental abnormalities including generalized thin hypoplastic amelogenesis imperfecta, intrapulpal calcifications and delay of tooth eruption. It has been described in the beginning in four patients from a consanguineous family by Martelli et al. One of the patients presented with intellectual deficit. O’Sullivan et al. identified recently a homozygous nonsense mutation in exon 2 of the *FAM20A* gene in a family with AIGFS [15].

The *FAM20A* gene (family with sequence similarity 20, member A) is located on chromosome 17q24.2, and consists of 11 exons; the protein FAM20A, 541 aminoacids, is expressed in ameloblasts and gingivae. FAM20A plays a fundamental role in enamel development and gingival homeostasis.

Six *FAM20A* mutations were reported in patients with AIGFS (Table 1). The mutation c.406C > T (p.Arg136Ter) in exon 2, was identified by autozygosity mapping combined with exome sequencing, in a consanguineous family [15].

**Table 1:** **Table summarizing different mutations reported in the** ***FAM20A*** **gene and their associated phenotype in patients with AIGFS**

Mutation	Phenotype	Reference
c.406C > T (p.Arg136Ter)	Thin generalized hypoplastic AI, intrapulpal calcifications, delayed tooth eruption, failure of tooth development	O’Sullivan et al. [15]
	Mental retardation	
c.34_35delCT	Generalized hypoplastic enamel , failed eruption of permanent teeth with dilaceration of the root, severe generalized gingival hyperplasia, agenesis of the left mandibular second premolar	Cho et al. [16]
c.34_35delCT	Hypoplastic AI affecting primary and permanent dentition, generalized gingival hyperplasia, the crowns were short, yellow-brown, and covered with little or no enamel, supra-incisive diastema.	Our case
c.813-2A > G	Generalized hypoplastic enamel failed eruption of permanent teeth with dilaceration of the root, mild localized gingival hyperplasia, especially in the anterior maxillary region.	Cho et al. [16]
c.1175_1179delGGCTC	Hypoplastic enamel , failed eruption of permanent teeth with dilaceration of the root, and mild localized gingival hyperplasia, especially in the anterior maxillary region.	Cho et al. [16]
c.590-2A > G	Generalized hypoplastic enamel failed eruption of permanent teeth with dilaceration of the root, mild localized gingival hyperplasia	Cho et al. [16]
c.826C > T		

More recently, Cho et al. identified three homozygous mutations (c.34_35delCT, c.813-2A > G, c.1175_1179delGGCTC) in three families and a compound heterozygous mutation (c.[590-2A > G] + [c.826C > T]) in one family with AIGFS, with different ethnical origin [16].

Little is known about the consequences of mutations in *FAM20A* gene. O’Sullivan et al. speculated that the position of the termination codon in exon 2 is most likely to result in nonsense-mediated degradation of the mutant transcript with a consequent loss of FAM20A protein [15].

The mutation identified in our patient (c.34_35delCT) and reported by Cho et al. in 2012, is predicted to result in a frame shifts and premature termination codon. This case reported by Cho et al. in 2012 had no extra-oral syndromic features, displayed generalized hypoplastic enamel with yellowish hue, delayed or failed eruption of permanent tooth with dilaceration of the root, severe gingival hyperplasia and agenesis of the left mandibular second premolar. There is almost no clinical difference between this patient reported carrying the mutation c.34_35delCT and our patient carrying the same mutation (Table 1); our patient has hypoplastic AI affecting primary and permanent dentition, a generalized gingival hyperplasia and absence of many teeth. However, Cho et al. noted that the mandibular second premolars are commonly involved in tooth agenesis and suggested that the tooth agenesis was unrelated to the *FAM20A* mutation.

AIGFS cases were reported by different countries all over the world. To our knowledge, this is the first one reported a Moroccan patient with AIGFS. Here, we describe the clinical and genetic data of a Moroccan patient with AIGFS syndrome. This diagnosis allowed us to provide an appropriate management to the patient, to make a genetic counseling to her family, and to enrich genetic data on Moroccan population. We suspect that the prevalence of AIGFS in Morocco might be high; especially with the high rate of consanguinity in Morocco (15.25%), this leads to an increased level of autosomal recessive diseases [17].

## Conclusion

To conclude, our results will help clinicians and molecular geneticists to determine the genetic etiology of AI patients. We suggest that mutations of *FAM20A* gene are causative for AIGFS and underline the recurrent character of the c.34_35delCT in two different ethnic groups.

## Consent statement

Written informed consent was obtained from the patient’s legal guardian for publication of this Case report and any accompanying images. A copy of the written consent is available for review by the Editor of this journal.

### Availability of supporting data

The data sets supporting the results of this article are included within the article.

## Abbreviations

AI: Amelogenesis imperfecta
AIGFS: Amelogenesis imperfecta and gingival fibromatosis.
